# COLLABORATIVE RESEARCH DEVELOPMENT: ESTABLISHING A RESEARCH-AGENDA AT A UNIVERSITY-BASED RETIREMENT COMMUNITY

**DOI:** 10.1093/geroni/igae098.2019

**Published:** 2024-12-31

**Authors:** M Aaron Guest, Molly Maxfield, Lindsey Beagley, Sigourney Schaffer, Becky Owens, David Coon

**Affiliations:** Arizona State University, Phoenix, Arizona, United States; Arizona State University, Phoenix, Arizona, United States; Arizona State University, Tempe, Arizona, United States; Arizona State University, Tempe, Arizona, United States; Arizona State University, Tempe, Arizona, United States; Arizona State Unviersity, Phoenix, Arizona, United States

## Abstract

University-Based Retirement Communities (UBRCs) represent a novel trend toward creating age-inclusive environments. These emerging housing options for older individuals generally function as continuing care retirement communities but stand out due to their close ties and university integration. They strive to foster intergenerational exchanges and offer older adults the chance to engage in university life and community. At Arizona State University, Mirabella at ASU is uniquely situated at the heart of the Tempe, AZ Campus, and is one of the few UBRCs physically located on a campus. Mirabella’s integration into the university has opened doors for collaborative research projects that add to broadly the nascent literature on UBRCs and aging. Residents expressed interest early in partnering with researchers to understand more about the UBRCs and aging in a university setting. To further these goals, a joint panel of residents and researchers was formed to pinpoint mutual research interests. This initial gathering prompted a series of focus groups to fine-tune topics, eventually leading to an annual resident health survey and the establishing of a Community Research Advisory Board. Transitioning from idea to implementation required navigating complex considerations beyond those typically encouraged in community-based research. In this presentation, we will share our experience in developing a collaborative research partnership while navigating the at-times competing interests of the researchers, university, operators, and residents. Furthermore, with the increasing prevalence of these communities, we offer guidance on sustaining engagement and sharing best practices derived from our experiences.

